# Challenging Diagnosis: Babesiosis With a Coincidental Intraabdominal Mass

**DOI:** 10.7759/cureus.69348

**Published:** 2024-09-13

**Authors:** Panisara Fangsaard, Pavel Bleik, Kannate Chotiphansiri, Edward Bischof

**Affiliations:** 1 Internal Medicine, Bassett Medical Center, Cooperstown, USA

**Keywords:** babesia, babesiosis, diagnostic challenge, splenic infarction, tick-borne disease

## Abstract

Babesiosis is a tick-borne disease caused by an intraerythrocytic parasite known as *Babesia *spp. Its clinical presentation is often nonspecific and can range from asymptomatic to life-threatening, depending on the host’s immune status. This myriad of manifestations potentially overlaps with other familiar conditions such as hematologic malignancy. Fortunately, despite the variability of presentations, babesiosis can be effectively diagnosed by using simple and cost-effective methods like peripheral blood smears. Recognizing diverse presentations of babesiosis is crucial for accurate diagnosis and timely management. In this case, we highlight the importance of avoiding diagnostic anchoring and including babesiosis in the differential diagnosis, particularly in endemic areas and in the setting of uncommon complications such as splenic infarction.

## Introduction

Babesiosis is an emerging tick-borne infection caused by an intraerythrocytic parasite called *Babesia (B.)* spp. More than a hundred species of babesiosis have been identified but only a few, including *B. microti, B. divergens, B. duncani*, and a currently unnamed strain designated MO-1, can cause human babesiosis. Cases of *B. microti* infection have been reported with increased frequency globally, particularly in the Northeastern and Midwestern United States [1]. The disease manifestation can vary based on the host’s immunological status, ranging from asymptomatic to life-threatening [2].

Mild babesiosis usually manifests with a wide range of nonspecific symptoms such as fever, sweats, fatigue, and myalgia. Several factors, including asplenia and older age, have been associated with severe presentations [3]. Previous studies have reported complications involving neurologic, cardiologic, hematologic, and splenic systems. Neurologic complications include confusion, delirium, and impaired consciousness [4] while atrial fibrillation is identified as the most common cardiac issue [5]. Additionally, uncommon but potentially life-threatening complications, such as hemophagocytic lymphohistiocytosis (HLH) and splenic infarction or rupture, have been noted [6,7]. The splenic rupture could be the first manifestation of the disease [8].

Therefore, diagnosing babesiosis remains challenging due to its nonspecific and varied presentations, including uncommon complications that may overlap with other diseases, especially cancer. In this case, we demonstrate the challenge of diagnosing babesiosis in the setting of splenic infarction and an intraabdominal mass.

## Case presentation

**Figure 1:**
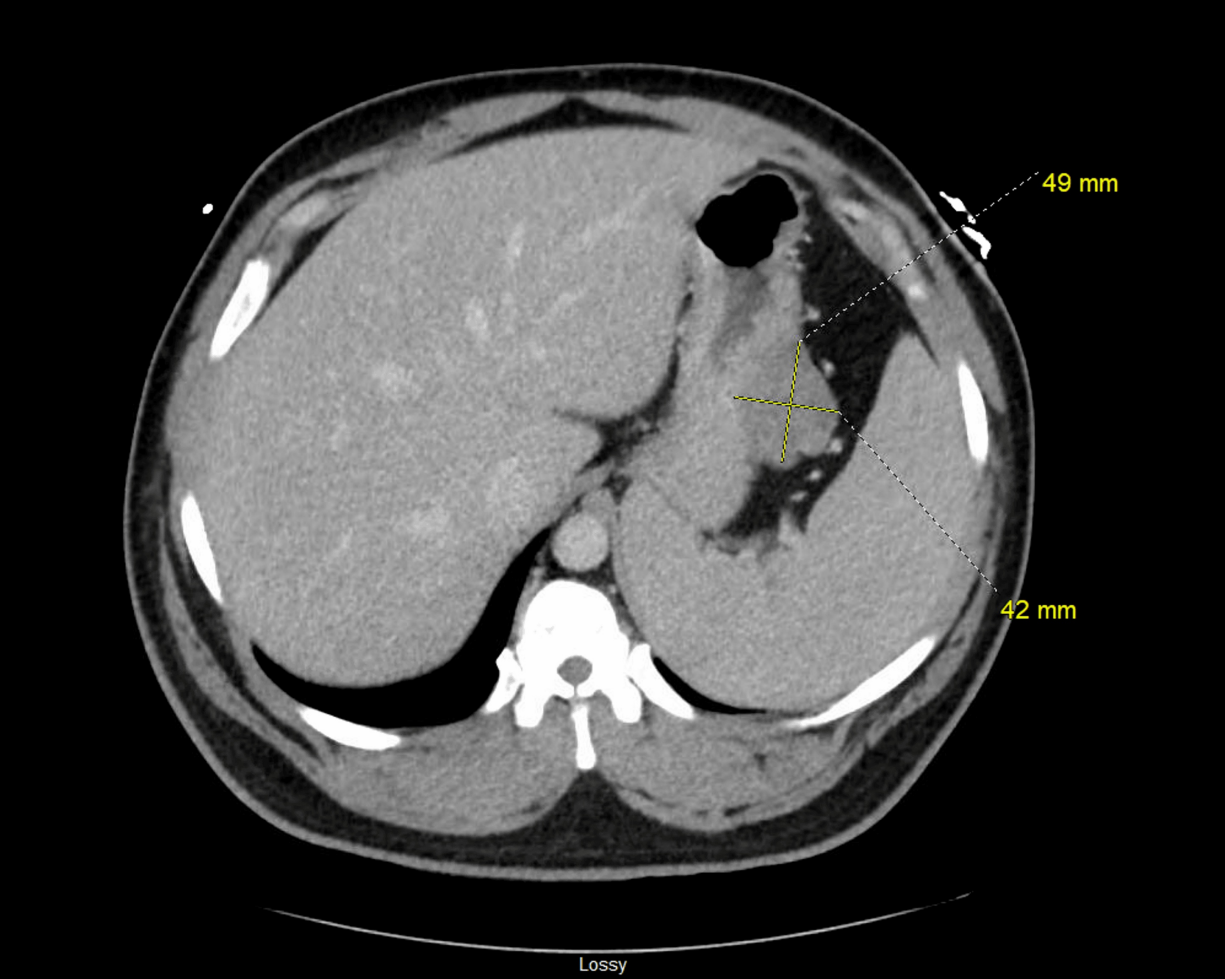
CT scan abdomen with IV contrast revealed a soft tissue mass of 5.6x2.9x6 cm abutting the posterolateral wall of the body of the stomach

**Figure 2:**
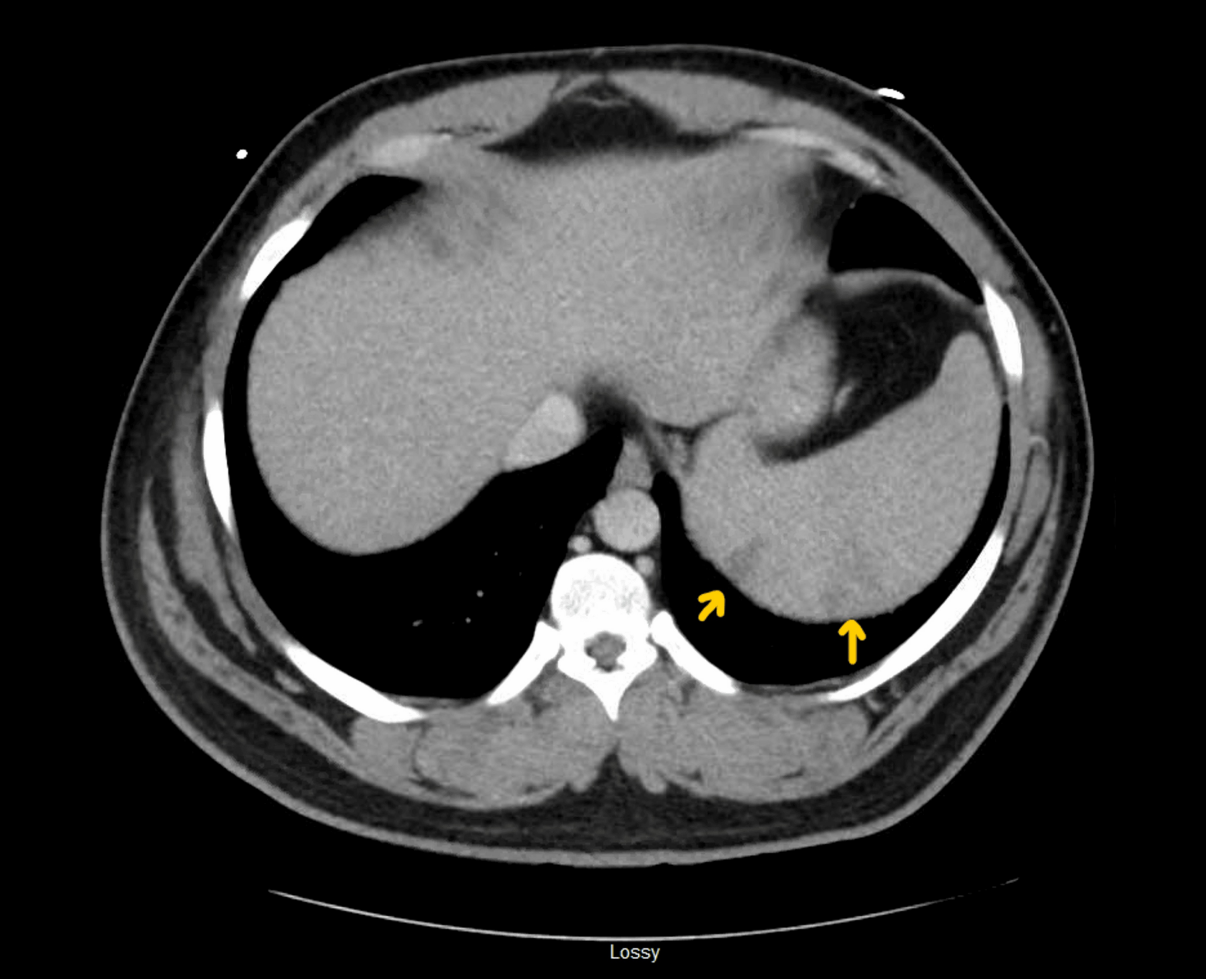
CT scan abdomen with IV contrast revealed splenomegaly with wedge-shaped infarcts

**Figure 3:**
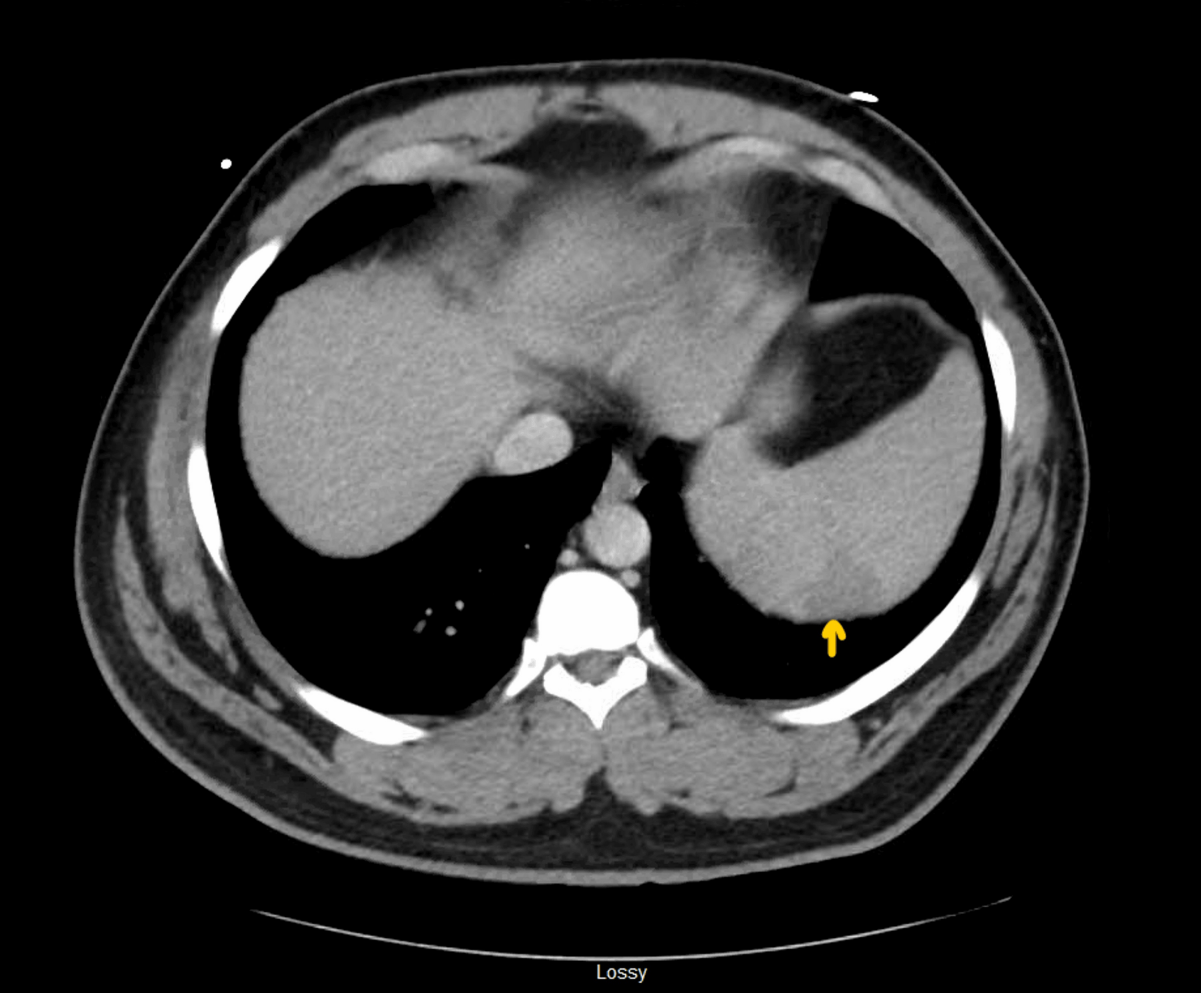
CT scan abdomen with IV contrast revealed splenomegaly with a wedge-shaped infarct

A 40-year-old male with no significant past medical history presented with a chief complaint of fever for two weeks. The patient reported intermittent fever, chills, drenching sweats, and extreme fatigue. His symptoms were accompanied by left-sided abdominal pain. He denied experiencing diarrhea, urinary or respiratory symptoms, or significant weight loss. He spent his summer doing a lot of outdoor fishing in the Northeast of the United States; however, he did not notice a tick bite. Vitals showed an elevated temperature of 38.6 °C, blood pressure of 142/78 mmHg, pulse 108/minute, respiratory rate of 21/minute, and oxygen saturation (SpO2) of 95% on room air. The physical examination was negative for hepatomegaly or abdominal mass, but mild splenomegaly was noted. Laboratory results revealed leukocyte 5,700 cells/L, hemoglobin 12.4 g/dL, platelet 63,000 cells/uL, lactate dehydrogenase (LDH) 467 U/L, and normal liver function tests and lipase. Computed tomography (CT) of the abdomen and pelvis with intravenous contrast demonstrated splenomegaly with three wedge-shaped infarcts and a soft tissue mass measuring 5.6x2.9x6 cm abutting the posterolateral wall of the body of the stomach (Figures 1-3). At this point, hematologic malignancy, particularly lymphoma, was high on the differential diagnosis due to the presence of fever, drenching sweats, elevated lactate dehydrogenase (LDH), splenic infarcts, and an intraabdominal mass.

**Figure 4:**
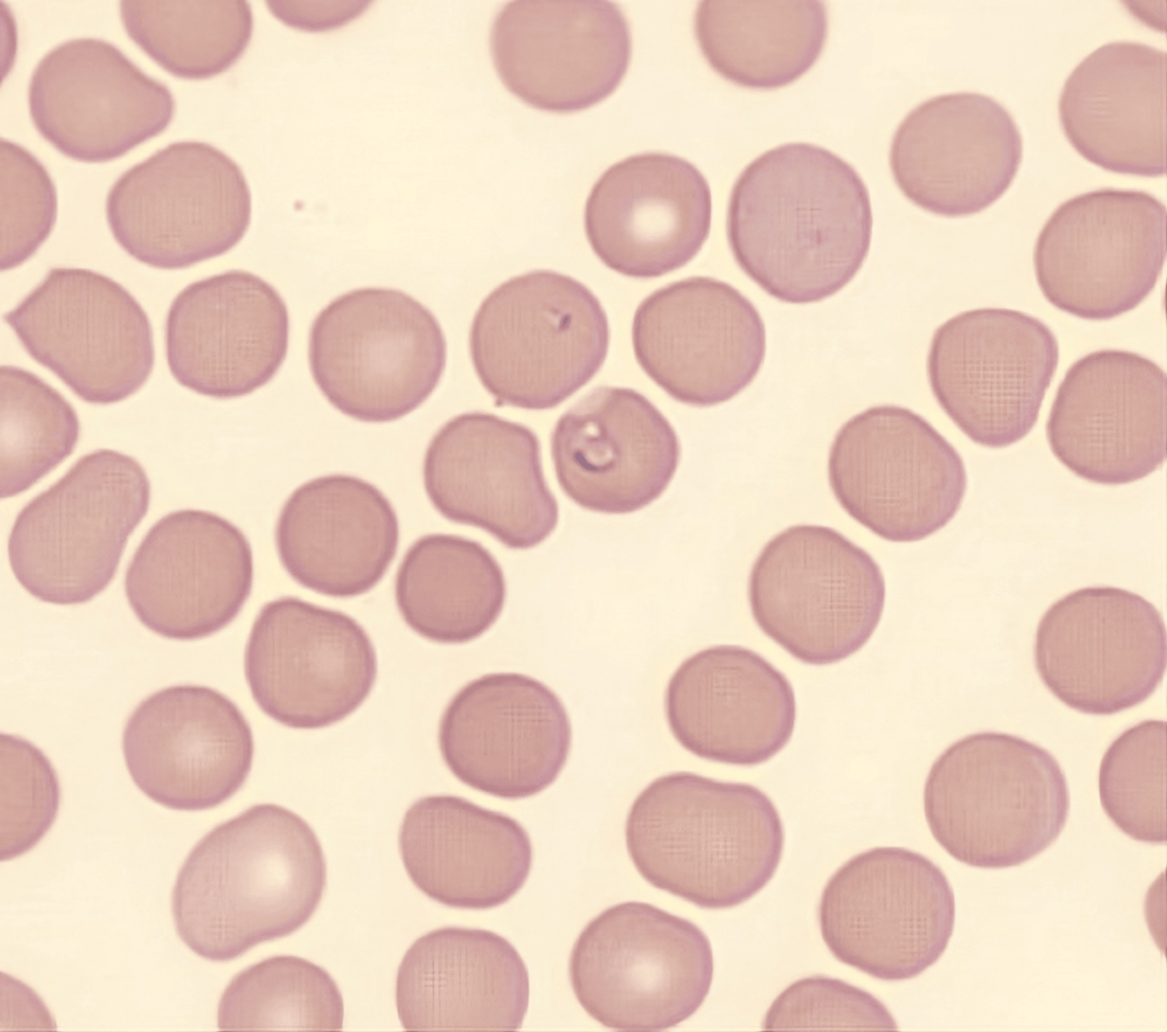
PBS showed ring-form parasites within red blood cells

The decision was made to pursue an abdominal mass biopsy to determine the etiology of his illness. However, considering an invasive procedure in the setting of thrombocytopenia, the transitioning team decided to reevaluate for potential alternative causes and broaden the differential. A peripheral blood smear (PBS) was obtained and revealed the presence of ring-form parasites within red blood cells (Figure 4) with 1.2% *Babesia* species. Polymerase chain reaction (PCR) confirmed *Babesia microti* infection and was negative for other tick-borne diseases, including Anaplasmosis, Ehrlichiosis, and Lyme disease. Azithromycin (500 mg oral once daily) and atovaquone (750 mg oral every 12 hours) were started, resulting in an improvement of his symptoms and thrombocytopenia. Splenic infarction was managed with conservative treatment, including intravenous fluids and pain control.

The patient was discharged home after a three-day hospitalization and later completed a 10-day course of antibiotics. He underwent an abdominal biopsy as an outpatient after thrombocytopenia improved. The abdominal mass’s pathology and immunohistochemistry results revealed bland epithelioid spindle cells with positive CD117 and DOG1. Gastrointestinal stromal tumor (GIST) was diagnosed with peritoneal metastasis, based on a positive emission tomography scan (PET). He underwent laparoscopic tumor resection followed by adjuvant imatinib.

## Discussion

Clinical physicians should maintain awareness of diagnostic anchoring when gathering clinical information to form a diagnosis, especially in diseases with myriad presentations. Babesiosis is a multifaceted disease that can manifest with a broad spectrum of symptoms influenced by the host’s immunological status [3]. This variability in presentation underscores the challenge of accurately diagnosing Babesiosis, particularly in patients with concurrent unknown medical diseases. The occurrence of fever, drenching sweats, a newly detected abdominal mass, and splenic infarcts heightened concern for a hematologic malignancy such as lymphoma. When cancer is on the differential, it can draw one’s attention away from other considerations and potentially lead to cognitive bias.

Anchoring bias is reported as one of the most prevalent cognitive errors in medicine. It occurs when physicians form an early assumption on initial information, even when they encounter new or conflicting evidence. Broad differentials and reassessing new clinical information on the original diagnosis should be pursued to mitigate fixating on a single potential diagnosis [9]. In this case, although the patient presented with clinical pictures suggestive of lymphoma, other potential causes of hemolytic anemia and splenic infarction were worth evaluating. Furthermore, the patient’s epidemiological background, Northeastern United States, should raise concern for possible tick-borne diseases [3].

With babesiosis on the differential and concern about an upcoming invasive procedure in the setting of thrombocytopenia, a PBS was examined. It is known to be a basic, timesaving, and cost-effective diagnostic tool that can help narrow down the differential and facilitate timely treatment. Nevertheless, it is worth noting that utilizing PBS as a diagnostic tool has some limitations. In the convalescent and early stages of infection, where parasitemia is usually less than 1%, diagnosis via PBS may fail [10]. Therefore, more sensitive investigations are often required. PCR assays can detect early or low-grade infections [2]. This patient also underwent PCR testing, which subsequently confirmed babesiosis infection.

Another pertinent finding in this patient was splenic infarction. It has been reported to be commonly related to hematologic disorders and malignancy, blunt abdominal trauma, hypercoagulable state, and embolic illness [11]. Based on the literature review, no research has documented an association between GIST and splenic infarction to date. However, it is known that an abdominal tumor can cause splenic infarction if it compresses the splenic artery [12]. Splenic infarction in babesiosis is uncommon but worth considering. Previous studies report splenic complications in younger and healthier patients with a low burden of parasitemia, as seen in this case [13-15]. Splenic complications, including infarction and rupture, likely result from a robust cellular immune response and splenic function in young, healthy patients, leading to pathological strain from splenic red cell clearance [13].

In summary, our patient concurrently dealt with two illnesses: babesiosis and GIST. Babesiosis should be considered in the differential diagnosis when a patient in an endemic area presents with nonspecific symptoms and evidence of hemolytic anemia. Physicians should remain vigilant against diagnostic anchoring bias, which can lead to premature closure on a single diagnosis. Prompt management with atovaquone and azithromycin effectively treated babesiosis in this patient and led to improvement in his symptoms and thrombocytopenia.

## Conclusions

Babesiosis can present with a broad spectrum of symptoms that potentially overlap with other conditions, including less common findings such as splenic infarction. Clinicians should maintain a high level of awareness to include babesiosis in the differential diagnosis and utilize PBS to assist in timely diagnosis. The learning point from this case highlights the importance of avoiding anchoring bias and underscores the possibility of concurrent diseases. This nuanced decision-making would assist in providing comprehensive care, prompt management, and sparing patients from invasive procedures during non-emergency situations.

## References

[1] Babesiosis (2024 (2024). Babesiosis. Centers for Disease Control and Prevention. https://www.cdc.gov/dpdx/babesiosis/index.html.

[2] Waked R, Krause PJ (2022). Human babesiosis. Infect Dis Clin North Am.

[3] Vannier EG, Diuk-Wasser MA, Ben Mamoun C, Krause PJ (2015). Babesiosis. Infect Dis Clin North Am.

[4] Locke S, O'Bryan J, Zubair AS, Rethana M, Moffarah AS, Krause PJ, Farhadian SF (2023). Neurologic complications of babesiosis, United States, 2011-2021. Emerg Infect Dis.

[5] Spichler-Moffarah A, Ong E, O'Bryan J, Krause PJ (2023). Cardiac complications of human babesiosis. Clin Infect Dis.

[6] Akel T, Mobarakai N (2017). Hematologic manifestations of babesiosis. Ann Clin Microbiol Antimicrob.

[7] Sahu KK, Petrou N, Cohn Z, Bathini V (2020). Splenic sequela of babesiosis. QJM.

[8] Dumic I, Patel J, Hart M, Niendorf ER, Martin S, Ramanan P (2018). Splenic rupture as the first manifestation of Babesia microti infection: report of a case and review of literature. Am J Case Rep.

[9] Norman GR, Eva KW (2010). Diagnostic error and clinical reasoning. Med Educ.

[10] Meredith S, Oakley M, Kumar S (2021). Technologies for detection of Babesia microti: advances and challenges. Pathogens.

[11] Chapman J, Helm TA, Kahwaji CI (2023). Splenic Infarcts.

[12] Yazici P, Kaya C, Isil G, Bozkurt E, Mihmanli M (2015). Splenic infarction - a rare cause of acute abdominal pain following gastric surgery: a case series. Int J Surg Case Rep.

[13] Patel KM, Johnson JE, Reece R, Mermel LA (2019). Babesiosis-associated splenic rupture: case series from a hyperendemic region. Clin Infect Dis.

[14] Dumic I, Madrid C, Rueda Prada L, Nordstrom CW, Taweesedt PT, Ramanan P (2020). Splenic complications of Babesia microti infection in humans: a systematic review. Can J Infect Dis Med Microbiol.

[15] Sung LH, Sundaram AH, Glick AL, Chen DF, Shipton L (2021). Babesiosis as a cause of atraumatic splenic injury: two case reports and a review of literature. J Gen Intern Med.

